# Transgene-induced cell death following dengue-2 virus infection in *Aedes aegypti*

**DOI:** 10.1038/s41598-023-32895-9

**Published:** 2023-04-12

**Authors:** Danilo O. Carvalho, Andre L. Costa-da-Silva, Vivian Petersen, Micael Santana de Souza, Rafaella S. Ioshino, Isabel C. S. Marques, Alexander W. E. Franz, Ken E. Olson, Anthony A. James, Margareth L. Capurro

**Affiliations:** 1 Department of Parasitology, Institute of Biomedical Sciences, University of São Paulo, São Paulo, SP 05508 Brazil; 2 Department of Veterinary Pathobiology, College of Veterinary Medicine, University of Missouri, Columbia, MO 65211 USA; 3 Center for Vector-Borne Infectious Diseases (CVID), Department of Microbiology, Immunology, and Pathology, Colorado State University, Fort Collins, CO 80523-1685 USA; 4 Department of Microbiology & Molecular Genetics, University of California, Irvine, CA 92697 USA; 5 Department of Molecular Biology & Biochemistry, University of California, Irvine, CA 92697 USA; 6 Instituto Nacional de Ciência e Tecnologia em Entomologia Molecular, Universidade Federal do Rio de Janeiro, Rio de Janeiro, Brazil

**Keywords:** Viral infection, Genetic engineering

## Abstract

Dengue viruses (DENVs) are mosquito-borne flaviviruses causing millions of human infections each year and pose a challenge for public health systems worldwide. *Aedes aegypti* is the principal vector species transmitting DENVs to humans. Controlling *Ae. aegypti* is difficult due to the abundance of breeding sites and increasing insecticide resistance in the vector populations. Developing new vector control strategies is critical for decreasing the disease burden. One potential approach is genetically replacing *Ae. aegypti* populations with vector populations highly resistant to DENV transmission. Here, we focus on an alternative strategy for generating dengue 2 virus (DENV-2) resistance in genetically-modified *Ae. aegypti* in which the mosquitoes express an inactive form of Michelob_x (Mx), an antagonist of the Inhibitor of Apoptosis (IAP), to induce apoptosis in those cells in which actively replicating DENV-2 is present. The inactive form of Mx was flanked by the RRRRSAG cleavage motif, which was recognized by the NS2B/NS3 protease of the infecting DENV-2 thereby releasing and activating Mx which then induced apoptosis. Our transgenic strain exhibited a significantly higher mortality rate than the non-transgenic control when infected with DENV-2. We also transfected a DNA construct containing inactive Mx fused to eGFP into C6/36 mosquito cells and indirectly observed Mx activation on days 3 and 6 post-DENV-2 infections. There were clear signs that the viral NS2B/NS3 protease cleaved the transgene, thereby releasing Mx protein into the cytoplasm, as was confirmed by the detection of eGFP expression in infected cells. The present study represents proof of the concept that virus infection can be used to induce apoptosis in infected mosquito cells.

## Introduction

The four serotypes of dengue virus (DENV-1-4; *Flaviviridae*; *Flavivirus*) continue to cause the most significant arboviral disease affecting human health worldwide. In 2019, 3.1 million cases were reported in the Americas, with more than 25,000 exhibiting severe disease and according to the Pan-American Health Organization (PAHO), a total of 2.3 million cases of dengue disease were reported in the same region in 2020^1–3^.

The yellow fever mosquito, *Aedes aegypti*, is the principal DENV vector. Females of this species also transmit other arboviruses, such as Zika virus (ZIKV; *Flaviviridae*; *Flavivirus*), chikungunya virus (CHIKV; *Togavivirdae*; *Alphavirus*), and yellow fever virus (YFV; *Flaviviridae*; *Flavivirus*), each causing recent outbreaks in the Americas in 2015, 2016 and 2019, respectively^4–6^. Arboviral diseases across the globe cause the death of hundreds of thousands of people annually and affect, economically and socially, millions of people emphasizing the urgent need for innovative approaches to suppress transmission of arboviruses by *Ae. aegypti*.^7–9^.

A novel vector control strategy, population replacement, is a self-sustaining approach that introduces a new genetic feature to a vector population to block its ability to transmit a pathogen of interest. This approach does not eliminate the vector species, thereby avoiding species loss and potentially associated environmental impacts^10,11^. The development of molecular tools during the last decades made it possible to produce genetically-engineered mosquitoes refractory to pathogen transmission^12–15^. Disease control would be based on the release of such genetically-modified strains in the field, where they would mate with local wild populations, which then would produce pathogen-resistant offspring. Over time, the wild population would be replaced with the refractory one, thereby reducing pathogen transmission to vertebrate hosts^16–18^. Fixation of a laboratory-engineered antipathogen effector gene into a field population would require the linkage of the effector to a gene-drive system such as those based on CRISPR/Cas9^19,20^. Previously, transgenic *Ae. aegypti* lines expressing anti-DENV effector genes as RNAi triggers have been generated, and this effector type significantly impaired virus replication and transmission^21–23^. Genetically-engineered mosquitoes expressing a polycistronic cluster of small synthetic RNAs targeting ZIKV were proven capable of blocking virus transmission^24^. In addition, transgenic mosquitoes expressing a DENV-targeting, single-chain variable fragment (scFv) originating from a human monoclonal antibody were refractory to all four DENV serotypes^25^.

**Figure 1:**
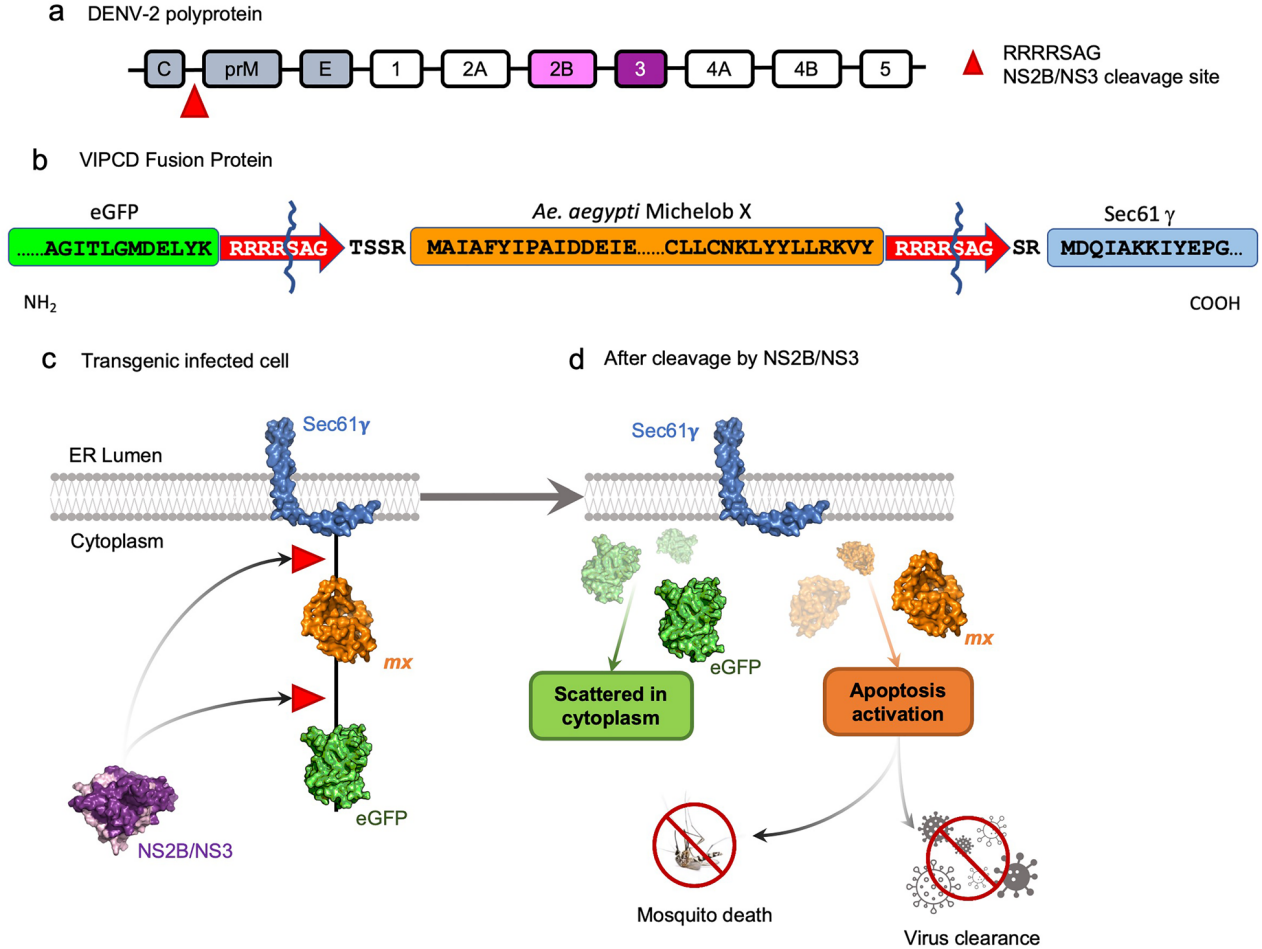
Genetic concept and mechanistic principle of the Virus Infection Promoting Cell Death (VIPCD) strategy. (**a**) General DENV polyprotein scheme showing the structural proteins C—Capsid; prM—Pre-membrane; E—Envelope; and nonstructural proteins NS1, NS2A, NS2B, NS3, NS4A, NS4B, NS5. The red triangle indicates the NS2B/NS3 cleavage site RRRRSAG releasing C (capsid) from the polypeptide of DENV-2. (**b**) The transgenic VIPCD fusion protein design with eGFP (enhanced green fluorescent protein) as a fluorescent marker; Mx—michelob_x ORF (antagonist of the Inhibitor of Apoptosis); Sec61γ—gamma subunit of the Sec 61 complex as a transmembrane domain that anchors the peptide in the endoplasmic reticulum. Shown is the partial amino acid sequence of the fusion protein surrounding *Ae. aegypti* Michelob x and the flanking NS2B/NS3 cleavage motifs (RRRRSAG) to release the recombinant Mx in the presence of DENV-2 NS2B/NS3. The wavy line indicates the amino acid position where NS2B/NS3-mediated cleavage is occurring. (**c**) DENV-2 infection of cells harboring the VIPCD fusion protein anchored to the endoplasmic reticulum (ER) membrane with the fluorescence signal close to the nucleus and Mx initially inactive. (**d**) The VIPCD mechanism is triggered by the activation of the NS2B/NS3 protease. The protease cleaves the viral polyprotein as shown in (**a**) as well as the RRRRSAG amino acid motif in the VIPCD fusion protein thereby releasing eGFP and Mx into the cytoplasm. Release of recombinant Mx then triggers a chain of events leading to cell death resulting in mosquito mortality and/or specific elimination of infected cells, eventually blocking viral transmission.

In the present study, we engineered *Ae. aegypti* to express an inactive form of the *michelob_x* (*mx*) gene product, Mx, an antagonist of the Inhibitor of Apoptosis (IAP)^26^, flanked by the amino acid motif RRRRSAG (targeted by the NS2B/NS3 protease of DENV-2^27^) and fused to a dominant marker, enhanced green fluorescent protein (eGFP) (Fig. 1a,b). This recombinant protein was tethered to the endoplasmic reticulum (ER) by the transmembrane region of the ER transport protein, Sec61-gamma^28,29^ (Fig. 1c). The strategy was to induce cell death through apoptosis following virus infection (*Virus Infection Promoting Cell Death*, VIPCD). NS2B/NS3, the protease of the infecting DENV-2, would catalyze the release of active *Mx* from the inactive recombinant protein, thereby promoting cell death via inhibition of IAP of the apoptotic pathway (Fig. 1d). Our transgenic line developed a higher DENV-2 titer, and a significantly higher mortality rate than the non-transgenic Higgs white eye (HWE) control strain following infection, likely due to increased induction of apoptosis. DENV-2 infected cells displayed a dispersed eGFP signal corresponding to the Mx fusion protein in the cytoplasm instead of the localized pattern seen in uninfected controls, consistent with a change in the localization due to the precursor cleavage by DENV-2 NS2B/NS3.

## Results

### VIPCD cell transfection and DENV-2 infection

**Figure 2:**
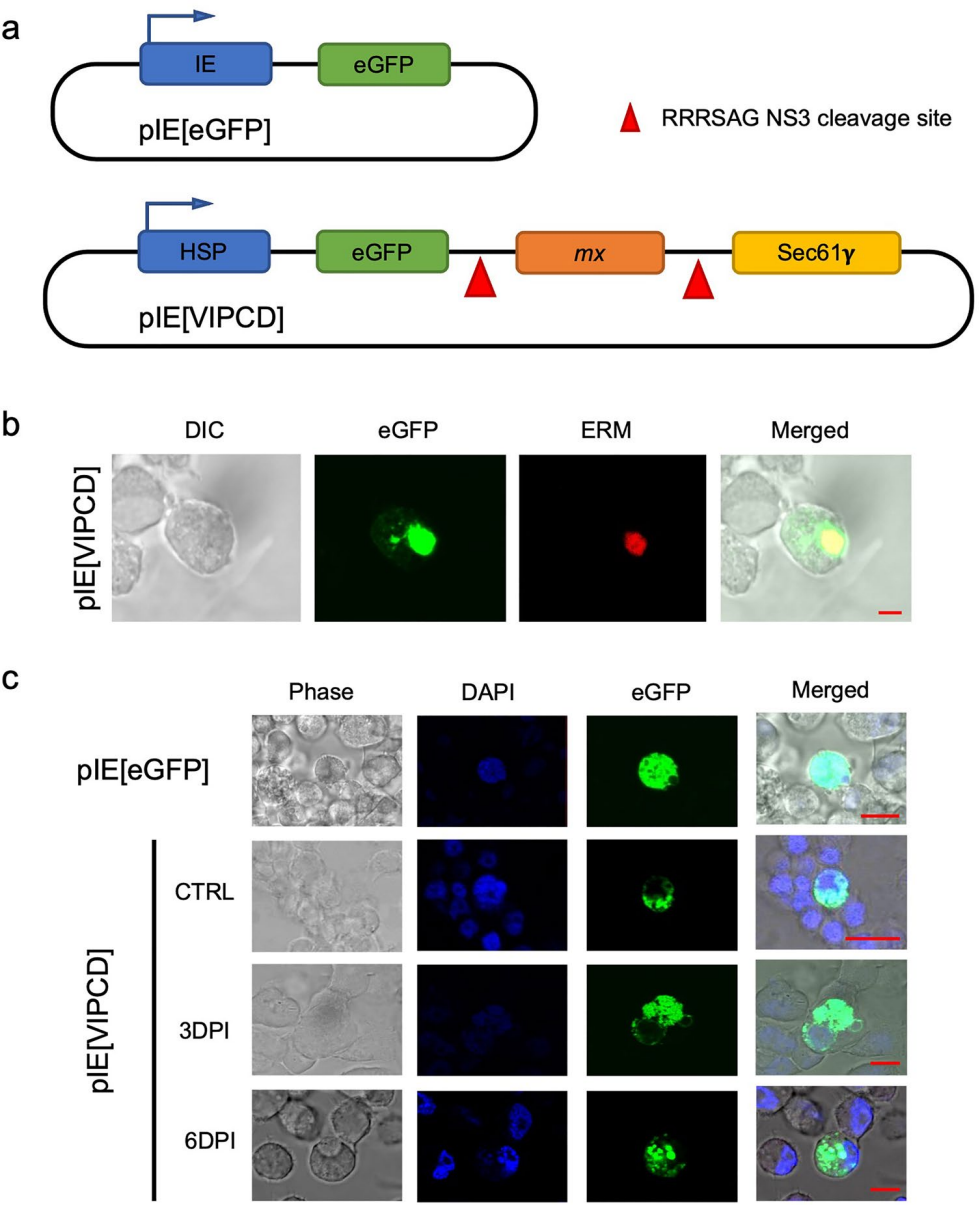
Co-localization of the VIPCD associated eGFP reporter at the endoplasmic reticulum of pIE[VIPCD] transfected C6/36 cells and release of the reporter following DENV-2 infection. (**a**) Schematic representation of the control pIE[EGFP] and pIE[VIPCD] plasmids used for the transfection of C6/36 cells. (**b**) Confocal images of C6/36 mosquito cells transfected with plasmid pIE[VIPCD] showing colocalization of the endoplasmic reticulum marker ERM and eGFP originating from VIPCD around the nuclear region of the cell. DIC, Differential Interference Contrast; eGFP, confocal image of eGFP expression; ERM, confocal image of endoplasmic reticulum marker expression; Merged, overlay of DIC, eGFP, and ERM. (**c**) Confocal images of eGFP expression in C6/36 cells transfected with plasmids pIE1[EGFP] or pIE1[VIPCD]; a non-infected control and pIE1[VIPCD] transfected cells following infection with DENV-2 (2.3 × 10^6^ PFU/mL) at 3 and 6 DPI are shown. Phase, phase-contrast visualization; DAPI, marker for nuclear DNA; merged, overlay of phase, DAPI, and eGFP.

The designed cell-death mechanism was validated initially in transfected C6/36 *Ae. albopictus* cells to examine the location and expression of the plasmid products and to indicate whether cell death was induced during virus infection. The pIE[VIPCD] construct differed from pMos[3xP3-DsRed-VIPCD] by the absence of the eye marker gene (3XP3-DsRed; Fig. 2a) in the former and the presence of a constitutive baculovirus immediate-early promoter to drive eGFP expression. The transfected pIE[VIPCD] C6/36 cells showed the presence of eGFP fluorescence in the region of the cell nuclei (Fig. 2b). To confirm that the recombinant protein was anchored in the ER with the help of Sec61γ, a specific ER marker (ERM) was used in the transfected cells and observed via confocal microscopy. ERM and eGFP co-localized at the expected site, validating the anchorage of the recombinant protein at the ER (Fig. 2b).

Following confirmation that the recombinant protein was localized as expected in non-infected control cells, the impact of infection was evaluated in cells exposed to DENV-2. eGFP was dispersed throughout the cytoplasm at 3- and 6-days DPI, while in the control non-infected cells, eGFP was confined around the nuclei as previously observed (Fig. 2b,c). During infection, cells also started showing evidence of apoptosis including DNA fragmentation and clustering, while DAPI marking in non-infected cells showed nuclear integrity. These data support the designed functionality of the mechanism proposed in this work, with DENV-2 infection triggering the cleavage of the recombinant protein.

### Establishment of transgenic VIPCD lines and their transgene expression levels

**Figure 3:**
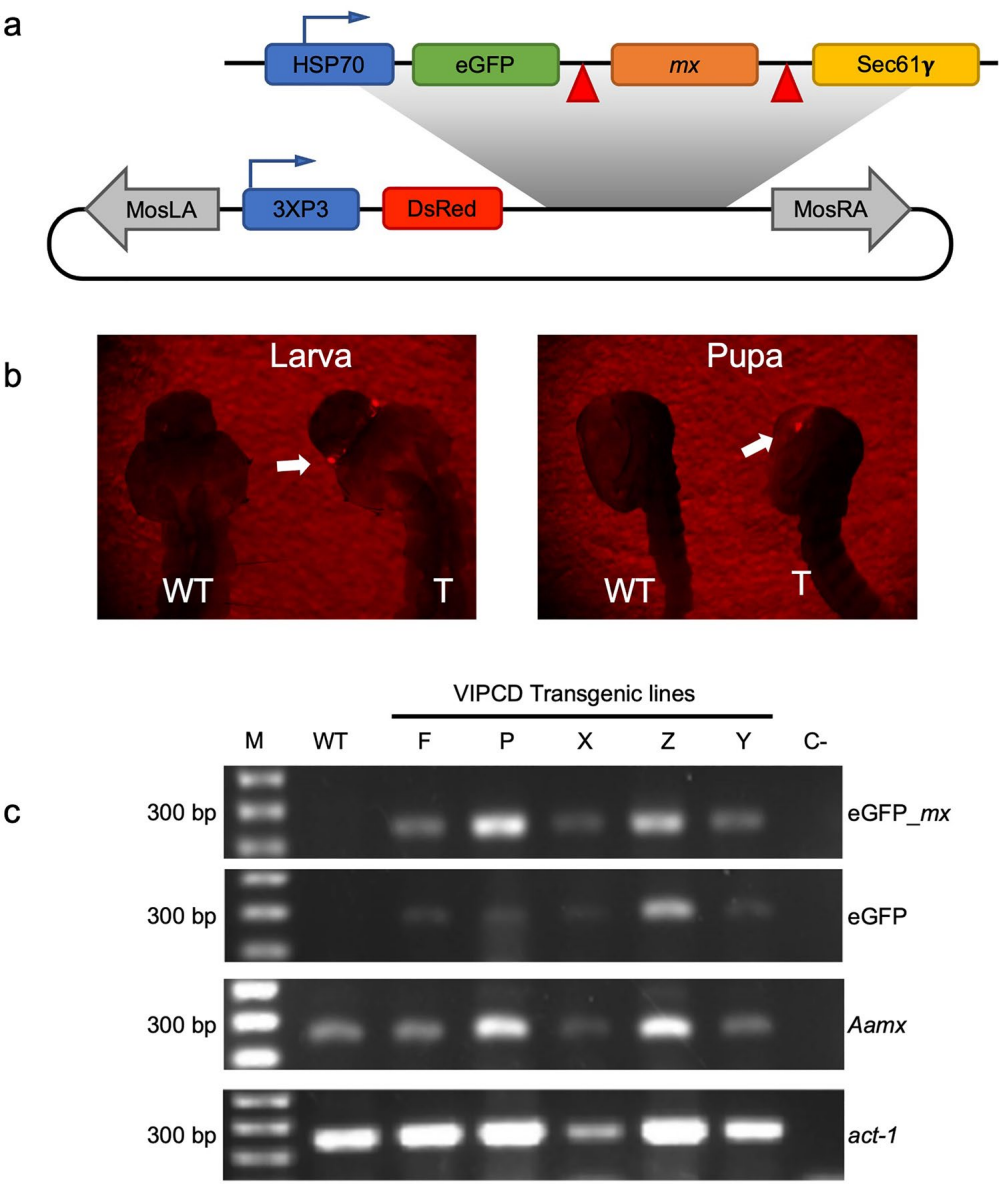
Description of VIPCD transgenic lines. (**a**) Schematic representation of the VIPCD construct subcloned into the transposon vector, pMos-DsRed (blue arrows indicate the promoter orientation) resulting in the donor plasmid pMos[3xP3-DsRed-hsp70-VIPCD]. HSP, heat-shock 70 promoter; eGFP, enhanced green fluorescent protein; red triangles, the RRRRSAG cleavage motif for NS2B/NS3; mx, michelob_x; Sec61γ, endoplasmic reticulum transmembrane domain. MosLA and MosRA, inverted terminal repeat sequences from the *mariner Mos1* transposable element; 3xP3, eye-specific promoter; DsRed, *Discosoma* red fluorescent protein expressed in the mosquito eye to identify transformants. (**b**) DsRed marker gene expression in *Ae. aegypti* (HWE) mosquitoes transformed with pMos[3xP3-DsRed-VIPCD]. Arrows point to the fluorescent protein expression in the eyes of transgenic larval and pupal stages under fluorescent light using a stereo microscope with a FITC-Texas Red filter. T, transgenic; WT (non-transformed HWE). (**c**) RT-PCR based expression profile of the VIPCD transgene in five transgenic lines (F, P, X, Z, and Y). The transcription profile of the VIPCD transgene was confirmed for all five transgenic lines by the amplification of expected fragments using specific primer sets to detect eGFP_mx (eGFP fused to mx) and eGFP. To confirm the integrity of cDNAs and the endogenous presence of mx, the endogenous Aamx and Actin-1 (Act-1) genes were amplified. M, molecular weight marker: 200 bp, 300 bp, and 400 bp.

One hundred fifty-three of the 2124 embryos (7.2%) co-injected with the donor (pMos[3xP3-DsRed-VIPCD]) and helper (pKhsp82MOS) plasmids survived to the adult stage. These G_0_ adults were outcrossed to the recipient HWE strain, and five transgenic lines were established based on DsRed expression in the eyes of G1 individuals (Table 1 and Fig. 3a,b). The designated transgenic lines F, P, X, Y, and Z were bred to homozygosity through repeated sibling matings. Lines were recognized as homozygous when 100% of all progeny expressed the DsRed eye maker in three consecutive generations.

**Table 1:** Microinjection of the pMos[3xP3-DsRed-VIPCD] construct into *Ae. aegypti* HWE preblastoderm embryos.

Construct	No. embryos injected	Survival %	No. screened larvae	No. transgenic lines	Transformation rate
pMos[3xP3-DsRed-VIPCD]	2124	153 (7.2%)^a^	15,568	5	3.8%^b^

^a^Percentage of injected embryos that hatched into adults.

^b^Estimated percentage of independently transformed lines generated per fertile adult (assuming ~ 50% fertility).

Using total RNA extracted from pooled individuals of each line, VIPCD transcript accumulation was assessed via RT-PCR (Figs. 3c and S3). VIPCD transcripts were observed in all transgenic lines when using the EGFP_*mx* oligonucleotide primer pair to amplify a 300 bp cDNA fragment from the chimeric eGFP/*mx* sequence (Table S1), while no amplicon was obtained as expected from the HWE (WT) controls (Fig. 3c). Similar results were obtained when amplifying the eGFP gene using eGFP-specific primers. As positive controls, expression levels of the *Actin-1* and endogenous *mx* genes were assessed, and transcript accumulations were detected for all transgenic lines and controls. Taken together, these data confirm successful genomic insertion and expression of the introduced transgene in lines F, P, X, Z, and Y.

### Dengue infection of VIPCD mosquitoes via intrathoracic injection

Once *VIPCD* expression was confirmed, we evaluated the mortality rates of the transgenic lines following their challenge with DENV-2 by intrathoracic injection. In this artificial infection method, the virus is directly delivered into the hemolymph and thoracic tissues of females, avoiding the midgut infection barrier (MIB) and the midgut escape barrier (MEB)^30^ ensuring that all injected mosquitoes would develop a DENV-2 infection.

**Figure 4:**
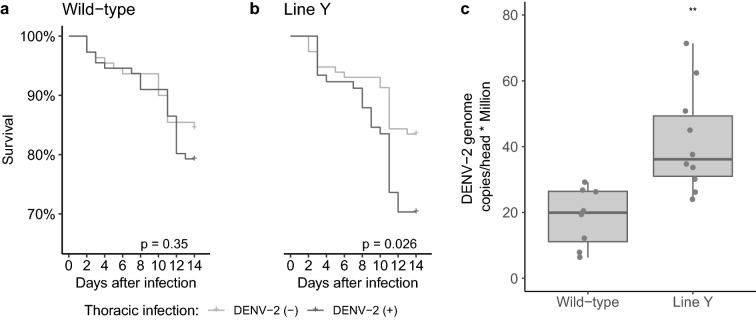
Survival and viral load of transgenic line Y following intrathoracic infection with DENV-2. (**a**) Kaplan–Meier survival curve based on the Log-rank test between the non-infected and infected control group (HWE strain, “wild-type”) and (**b**) the non-infected and infected transgenic line Y. Survival curves were performed for 15 days. Crosses represent censored data at the end of the lines. (**c**) Median number of DENV-2 RNA copies/head (per million) detected at 15 days DPI in control (HWE, “wild-type”) and line Y mosquitoes using the GLM procedure and the estimated marginal means (least squared mean), with Tukey contrasts multiple comparison method to determine statistical differences—boxplot represents the median, minimum and maximum, and 25% and 75% quartiles; points represent a scatter plot of their distribution.

The survival of HWE females injected with DENV-2 did not differ significantly (Chi^2^ = 0.9, df = 1, *p* > 0.05) in comparison to the control group comprising of females injected with virus-free cell culture supernatant (Fig. 4a), demonstrating that DENV-2 infections per se did not increase mortality in our experiments. However, survival rates of the DENV-2 injected females from the Y line differed significantly (Chi^2^ = 4.9, df = 1, *p* < 0.05) from those of control supernatant-injected females within the 15-day observation period (Fig. 4b). The Y line exhibited higher mortality (~ 10%) when infected with DENV-2 than the non-infected transgenic group. No statistical differences were observed between infected and non-infected mosquitoes of the other transgenic lines (Fig. S1).

We further assessed the DENV-2 RNA copy numbers/head of transgenic females following injection with cell supernatant containing DENV-2. Female heads of line Y showed a least-squared mean of 41.5 million viral RNA copies/head (with 34 million copies and 49.1 million copies—lower and upper confidence level, Fig. 4c). In comparison, the control had a mean of 18.6 million viral RNA copies/head (with 10.1 and 27 million RNA copies/head as lower and upper confidence levels). These results present a statistically significant difference, in which strain Y had a higher viral load than the control (Tukey contrasts multiple comparisons with z = 3.966 and *p* = 0.001). However, no statistical difference was observed in the number of DENV-2 RNA copies/head in the other transgenic lines (Fig. S2).

### Detection of apoptosis in the midguts of VIPCD mosquitoes

**Figure 5:**
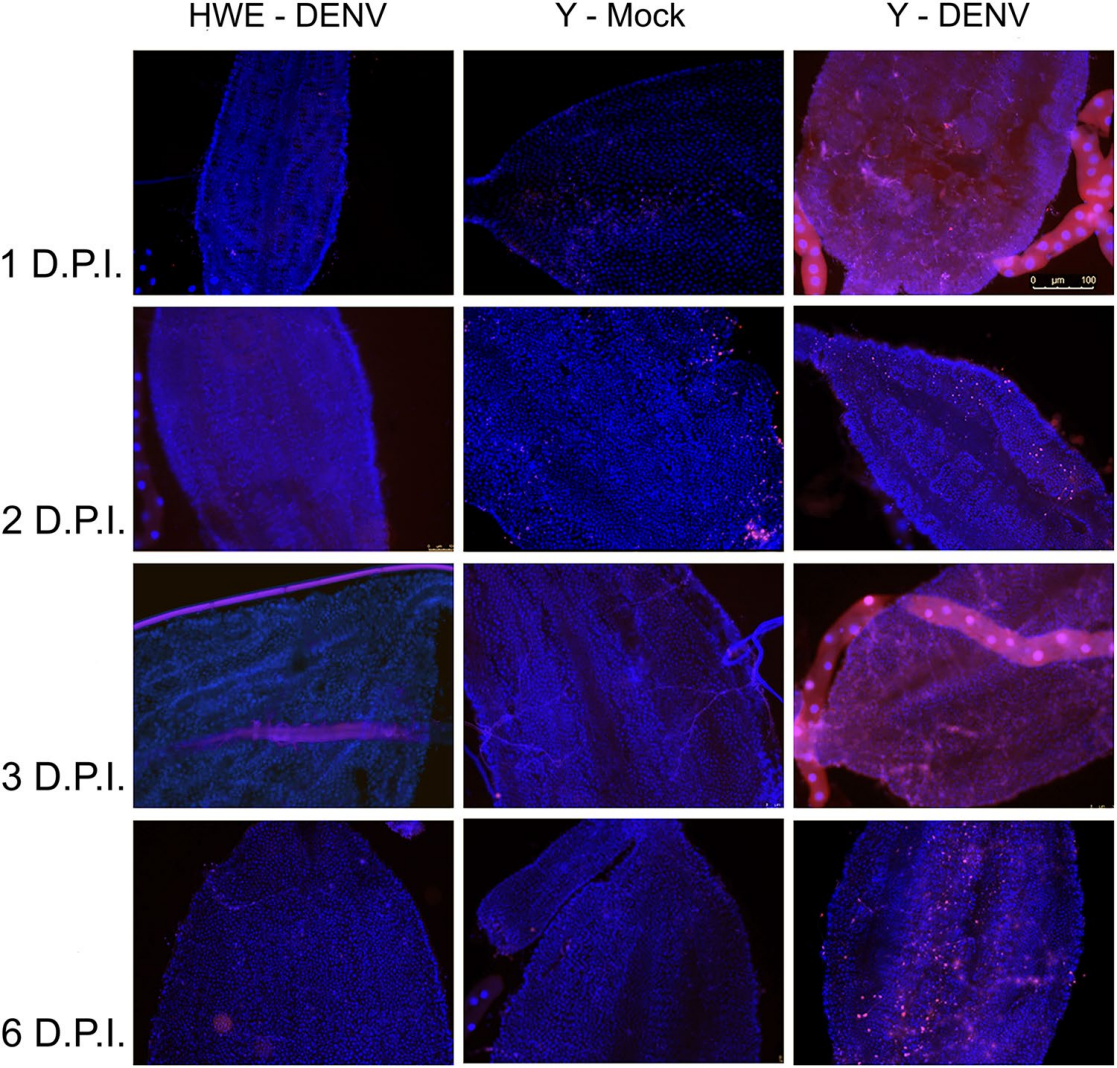
TUNEL staining of midguts from HWE (Higgs) and line Y females at 1, 2, 3, and 6 DPI following infection of mosquitoes with DENV-2 via intrathoracic injection. Counterstaining was performed using Hoechst 33342. Y-MOCK represents the Y line injected with cell culture supernatant containing no virus.

Based on the assessments of survival rates and DENV-2 replication, the induction of apoptosis was evaluated in midguts of mosquitoes infected with DENV-2 via intrathoracic injection at different time points post-infection. The TUNEL assay results showed that within the observed time course, the ten evaluated midguts of the non-transformed, DENV-2 injected HWE control strain showed no evidence of apoptosis or basal apoptotic events (Fig. 5). Similarly, when injected with a cell culture medium containing no virus, the Y line showed only minor basal apoptotic events at 2 days post-injection. By contrast, severe apoptosis was observed during the entire time course in the Y line when infected with DENV-2, indicating that this strain triggered the apoptotic cascade.

## Discussion

We developed a transgenic *Ae. aegypti* strain carrying a *Virus Infection Promoting Cell Death* (VIPCD) construct that was activated only in DENV-2 infected cells of the vector. The expression of viral N2B/NS3 protein triggered cell death by releasing a recombinant (pro-apoptotic) Mx protein into the cell's cytoplasm. Survival curves, quantitative RT-PCR, and confocal microscopy suggest that the products of the VIPCD construct activated the apoptotic pathway in the presence of DENV-2 without suppressing viral replication.

Our non-natural intrathoracic injection approach bypassed the midgut barrier, which could prevent or reduce the systemic infection of secondary tissues such as fat body, nerve tissue, ovaries, and salivary glands in the mosquito ^41^. By injecting the virus intrathoracically, we ensured that 100% of challenged mosquitoes were infected. Importantly, midgut infection rates were similar among the *Ae. aegypti* lines (wild-type and transgenics) due to the intrathoracic injection route. Thus, differences in viral replication efficiencies among the transgenic lines were likely responsible for the observed variable virus titers in the mosquitoes^31^.

The TUNEL assay showed induction of apoptosis during a time course in midgut tissue of line Y when infected with DENV-2. Furthermore, the survival rates of Y line females were significantly reduced when DENV-2 infected. By contrast, infected HWE control mosquitoes showed no signs of apoptosis and the intrathoracically-infected HWE mosquitoes had a similar survival pattern as the infected HWE. These observations indicate that the apoptotic cascade was initiated by the specific interaction between the pro-apoptotic effector and the replicating virus, thereby inducing increased mortality in infected individuals of line Y. The other strains (F, P, X, and Z) showed no significant differences in their DENV-2 head titers and survival rates relative to the controls. These observed phenotypic differences between line Y and the other four transgenic lines may be related to position effects due to transposon-mediated, quasi-random transgene integrations, which can lead to variable transgene expression and activation levels, thereby differentially affecting the overall fitness of the mosquito^32–35^.

The concept of heterologous gene (transgene) activation due to the replication activity of an arbovirus in mosquitoes has been previously explored for alphaviruses^36^. Instead of utilizing the viral protease and its recognition sequence motif for heterologous gene activation as shown here, the authors made use of the fact that alphaviruses such as Sindbis virus (*Togaviridae*; *Alphavirus*, SINV) are expressing a structural gene encoding sub-genomic RNA during their replication cycle. Mosquitoes were generated to harbor a *Mos1*-transposon based transgene containing the cDNA of the modified viral genome of SINV in which most of the nonstructural protein encoding genes were removed and all structural genes replaced by a fluorescent reporter. Only in the presence of the infecting homologous wild-type virus, reporter gene expression was observed in those mosquitoes.

Although our VIPCD transgenic lines showed the potential to reduce survivorship during DENV-2 infection, the observed mechanism appeared to be insufficient to completely block virus transmission by the mosquito or kill the infected transgenic mosquito quickly enough to prevent it from transmitting the virus. The released recombinant Mx may not have been fully active because it contained additional amino acid residues at its amino terminus (see Fig. 1b), which could have hampered further aminopeptidase processing of the protein required to reveal the IAP binding motif^37^. Based on those results, the virus would likely still be able to reach the salivary glands, and females would live long enough to infect a vertebrate host during their following ingestion of a blood meal^38^. Further studies should be conducted to assess the amount of virus a female could potentially transmit and evaluate its role in transmission since low virus loads do not always lead to a productive infection. Here we assessed the copy numbers of viral RNA, which is not necessarily a strong indicator for the quantity of infectious particles in a given tissue. It can be speculated that due to apoptosis, there may have been less infectious virus produced in our transgenic line as suggested when measuring the quantity of viral RNA^39^. However, the transgene construct should be further optimized so the system can more efficiently induce apoptosis and block viral transmission. As stated above, the overhanging N-terminal amino acid residues left attached to Mx following cleavage may have been a problem. It would be interesting to see if the NS2B/NS3 cleavage site can be re-engineered to allow the resulting peptide cleavage product to start with a methionine and still be functional.

Combining the results from the transfected cell culture and the transgenic mosquitoes, the present study represents a proof-of-concept showing that a wild-type virus can be used as a trigger for apoptosis inside infected cells. We are now characterizing the molecular events generated by DENV-2 infection in the transgenic lines to improve the system and achieve a more efficient VIPCD response. However, as the NS2B/NS3 cleavage motifs are rather specific for each flavivirus species^40^, it is not very likely that other viruses from this family would also induce an apoptotic response in our transgenic mosquito line as a consequence of the interaction between virus and transgene.

It is possible that population-replacement-based VIPCD or RNA-based approaches (i.e., RNAi to block DENV transmission) will lose efficacy over time due to the accumulation of mutations within the transgenes or reduction of transgene expression levels^41^. However, linking the antiviral effector to an efficient gene-drive system may allow a hypothetically targeted susceptible mosquito population to become virus-resistant before any deleterious mutations may accumulate and weaken the effector^13,42–45^.

## Conclusion

Here, a novel transgenic strategy to block DENV-2 transmission by mosquitoes has been developed as proof-of-principle. We show that it is possible to engineer and overexpress a transgene in *Ae. aegypti*, carrying an inactive form of the antagonist of the Inhibitor of Apoptosis, michelob-x (Mx), which gets activated in the presence of replicating DENV-2. During NS2B/NS3 mediated cleavage of the viral poly-protein the transgene-bound Mx was released, which then induced apoptosis and significantly increased mortality rates among those infected transgenic mosquitoes. Our concept shows potential and needs to be further evaluated and improved to achieve complete blockage of virus transmission.

## Material and methods

### Mosquito rearing

*Aedes aegypti* (Higgs white eye strain; HWE) mosquitoes were reared locally at the Institute of Biomedical Sciences, University of São Paulo, São Paulo (SP), Brazil. Mosquitoes were kept at 27 ± 1 °C, 75–80% relative humidity, and a 12∶12 h light:dark cycle. Larvae were fed on finely powdered fish food (Tetramin, Germany), and adult mosquitoes were maintained ad libitum on a 10% sucrose solution (w/v). Pre-mated five-day-old adult females were blood-fed using anesthetized mice when required***.***

### *Aedes albopictus* cell transfection and infection with DENV

C6/36 cells (2 × 10^6^) were plated in a T25 cell culture flask 1 day before transfection. Cells were transfected with pIE[VIPCD] (Figs. 1b and 2a) in which VIPCD was under control of a baculovirus immediate-early promoter using 25 μL Lipofectamine 2000 (Life Technologies, USA) at 5.0 μg plasmid per 2 × 10^6^ cells, and after three days, C6/36 cells were infected with DENV-2 (Jamaica 1409 strain; virus load was 2.3 × 10^6^ PFU/mL). On the 3rd and 6th days post-infection (DPI), eGFP fluorescence indicating cleavage and release of Mx into the cytoplasm during virus infection was monitored using confocal microscopy. The endoplasmic reticulum (ER) probe (ER-Tracker™ Red Invitrogen™) was used to detect and localize the ER and nuclear regions of each cell. Additionally, DAPI (Invitrogen™) was used to stain the nuclei. All imaging was performed using a Zeiss Laser Scanning Axiovert Confocal Microscope in the Infectious Disease Annex on the Foothills Campus of Colorado State University, applying the appropriate wavelength excitation filters to detect the emission wavelengths of the three different molecules/proteins and overlapping images.

### Plasmid construction

The chimeric VIPCD gene (Fig. 1b) was inserted into the pMos[3xP3-DsRed] transposon vector^46,47^ to yield pMos[3xP3-DsRed-VIPCD] (Fig. 3a), which was synthesized by Epoch Biolabs, Inc (TX, USA). The marker gene, DsRed, was placed under the control of the 3xP3 eye-specific promoter to identify transgenic individuals under a fluorescent microscope (Fig. 3b). The chimeric DENV-2 responsive construct contained the minimal promoter of the *heat shock protein 70* (*hsp70*) gene, resulting in constitutive gene expression throughout the mosquito life cycle. The expressed chimeric protein was anchored in the ER with the Gamma-subunit of the Sec61 protein^48^. Adjacent to the transmembrane protein was the Michelob_x protein (Mx), an Inhibitor of Apoptosis (IAP) antagonist, which is known to trigger apoptosis in *Ae. aegypti*^26^. The chimeric protein contained an eGFP fusion to track the cytoplasmic location of the cleaved protein form (Fig. 2a). Mx was flanked by the NS2/NS3 cleavage recognition motif RRRRSAG from DENV-2^49–51^.

### Mosquito embryo injection

Microinjections were performed as described^52^. Briefly, *Ae. aegypti* embryos were injected in the posterior pole with an injection solution containing 0.5 and 0.3 µg/µl of the *mariner Mos1-*based donor and transposase encoding helper plasmids, respectively, diluted in injection buffer (disodium phosphate 1 M and monosodium phosphate 1 M pH 6.8). The solution was injected using borosilicate glass needles in conjunction with an Eppendorf microinjection system, Femtojet, and TransferMan NK2 (Eppendorf, Germany). After microinjection, embryos were maintained in a humid chamber on moistened filter paper and water-soaked cotton for three days before hatching in a plastic tray with a ~ 2 mL larva diet. Female adults surviving injection (G_0_) were grouped into a maximum of 10 individuals and crossed with Higgs white-eye (HWE) control males in a 1:1 ratio. Injected males (G_0_) were maintained individually and crossed with ~ 10 HWE virgin females. For all crosses, mating occurred for at least 3 days, and females had access to a blood meal. Egg collection was performed as described previously^52^. Progeny (G_1_) larvae were screened for fluorescent eye marker expression. Transgenic individuals were outcrossed to HWE for another generation (G_2_) before being used in further experiments.

### DNA and RNA extraction and gene amplifications

Genomic DNA was extracted from pools of five mosquitoes/strain using the DNeasy Blood & Tissue kit (Qiagen). Conventional gene amplification (PCR) was used to assess the presence of VIPCD in the genome. Specific oligonucleotide primers were designed to detect eGFP fused to *mx* (eGFP_*mx*), eGFP, and endogenous *mx* (*Aamx*) (Table S1). In addition, a primer combination for the detection of *Actin-1* (*Act-1*) as an endogenous control was chosen. Total RNA was extracted from a pool consisting of five to seven-day-old HWE (WT) adult females (n = 10) and five transgenic lines (F, P, X, Z, and Y) using Trizol Reagent (Invitrogen™). Following DNaseI (Invitrogen™) treatment, first-strand cDNA synthesis was conducted for 30 min at 50 °C using 2 μg of treated total RNA and oligo(dT)12–18 primer in combination with SuperScript III (Invitrogen™). Conventional PCR was applied to amplify cDNAs encoding eGFP, endogenous *mx* (*Aamx*), eGFP fused to *mx* (eGFP_*mx*), and *Actin-1* (*Act-1*) as an endogenous control. The specificity of the reaction was confirmed when analyzing a negative control in which DNA or cDNA was replaced by water.

### DENV-2 amplification and detection

C6/36 (ATCC CRL-1660) cells were maintained in Leibovitz’s L-15 Medium (Gibco, Thermo Fisher) with 10% fetal bovine serum (FBS; Gibco, Thermo Fisher Scientific) and 1% antibiotic–antimycotic solution (Gibco, Thermo Fisher Scientific) at 28 °C. In addition, the cell culture line, LLC-MK2 (ATCC CCL-7), from *Macaca mulatta*, was maintained in Dulbecco's Modified Eagle Medium (DMEM) (Gibco, Thermo Fisher Scientific) supplemented with 5% FBS (Gibco, Thermo Fisher Scientific) at 37 °C with 5% CO_2_. These two cell types were used for the propagation and viral titration of DENV-2, respectively. Culture supernatant containing DENV-2 particles from ACS46 strain^53^ was kindly provided by the Evandro Chagas Institute (Belém, Brazil) and used to perform the intrathoracic infections of mosquito females (transgenic and non-transgenic). The (negative) injection control consisted of cell culture supernatant without virus.

### DENV-2 intrathoracic infection

Groups of 35 to 50 females were injected intrathoracically with ~ 276 nl of DENV-2 supernatant (viral load 7.0 × 10^6^ genomic copies) at 5–7 days post-emergence, using a Drummond Nanoinjector II (Drummond Scientific, BrookMall, Oakbrook, IL, USA) and a borosilicate capillary needle made with a needle puller (model P-97 Sutter Instrument, USA). Surviving females were maintained on 10% sucrose solution for 14 DPI (survival period). Females were then anesthetized with CO_2_ and kept on ice for head dissection and body collection. After removing the head, individual heads and bodies were immediately frozen in dry ice and stored at − 80 °C for molecular analysis.

### DENV-2 quantification by qRT-PCR

Total RNA was extracted from mosquito samples using the QIAmp Viral RNA Mini Kit (Qiagen, Valencia, CA, USA), and DENV-2 genomic copies were quantified using a one-step qRT-PCR as described previously^54^.

### Survival

Following the intrathoracic injections (with supernatants containing/not containing DENV-2), four groups of 25 infected females were observed for 15 days, based on the average time for the virus to reach the salivary glands and central nervous system^38^, and the number of dead mosquitoes recorded daily. The HWE strain was used as the non-transgenic control and subjected to intrathoracic injections with supernatants containing/not containing DENV-2.

### TUNEL assay

Three to 4 days post adult emergence, mosquitoes were microinjected intrathoracically with DENV-2 (ACS46) using the Nanoject II microinjector (Drummond Scientific Company). Three groups of 10 mosquitoes each were injected: (A) Y line mosquitoes injected with DENV-2; (B) Y line mosquitoes injected with Leibowitz LI 5 (GIBCO—New York, USA) as negative control; and (C) non-transformed HWE females injected with DENV-2. Each female received 69 nL/pulse accumulating a total of ~ 276 nl of virus during microinjection, and then was transferred to double-containment cages and housed in an NB2 insectary. Female midgut samples removed at 1, 2, 3, and 6 DPI were placed in Phosphate-Buffered Saline 1x (PBS 1x; pH 7.4) for a TUNEL assay using the Click-iT™ Plus TUNEL Assay Kit for In Situ Apoptosis Detection (Thermofisher Scientific). Briefly, dissected midguts were fixed onto glass slides by covering them with 4% paraformaldehyde for 15 min, washed, and subjected to a solution of 0.25% Triton™ X-100 in PBS 1× for 20 min, and washed once again. The samples were then covered with buffer TdT from the TUNEL kit and incubated for 10 min at 37 °C. The next step consisted of adding 50 µL TdT reaction buffer to the samples and incubation for 60 min at 37 °C. The TdT reaction buffer was removed, and 50 µL of Click-iT™ Plus TUNEL reaction cocktails were added and incubated for 30 min at 37 °C. The samples were washed, and 100 µL of 1X Hoechst 33342 fluorescent DNA stain was added for 15 min (for non-fragmented DNA), after which the stain was removed, and the slides with the midguts were mounted. The presence of apoptosis was visualized under a fluorescence microscope (Leica DMI6000B/AF6000, Leica, Germany) using an objective lens (10× and 40×) coupled to a digital camera system (DFC365FX, Leica), using fluorescent settings for Alexa Fluor 594, detecting DNA fragmentation. All images were rendered and processed using the Leica Application Suite X (Leica Application Suite X–LAS X. Software version 5.0.2).

### Ethics statement

The project was submitted to the Ethics Committee of Animal Usage from the Biomedical Science Institute of the University of Sao Paulo (CEUA-ICB USP) under the approved certificates 186, 187, 188/12/CEUA. The manuscript performed all methods in accordance with the regulations and accordance of the Ethics Committee. In addition, it follows the recommendations stated in the ARRIVE guidelines^55^.

### Statistical analysis

All analyses were performed using the R language in RStudio. A generalized linear model (*stats* R package) was used for all comparative studies considering alpha 0.05 and removal of outliers (identified using *boxplot*) according to the nature of the data; a binomial or Poisson distribution was used, depending on the type of data, percentages, and ratios for binomial or Poisson for counting. A Kaplan–Meier log-rank test was performed for survival analysis using the R packages *survival* and *survminer*^56,57^.

## Supplementary Information

Supplementary Information.

## Data Availability

The datasets generated during and/or analyzed during the current study are available from the corresponding author on reasonable request.
