# Supplementary material for: Transgene-induced cell death following dengue-2 virus infection in Aedes aegypti
Source: Sci Rep. 2023 Apr 12;13:5958. doi: 10.1038/s41598-023-32895-9 (PMC10097671; doi:10.1038/s41598-023-32895-9)
Supplement: Supplementary file 1 — Supplementary Information. [file 41598_2023_32895_MOESM1_ESM.pdf]

Supplementary Material

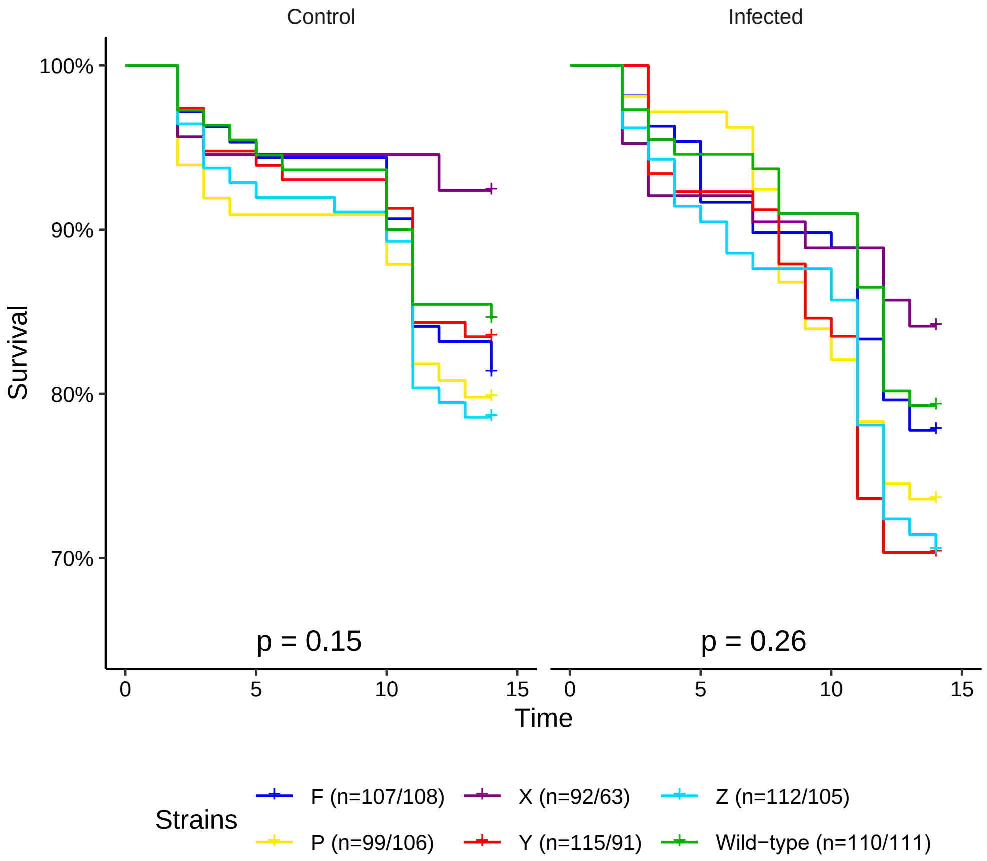

Figure S1. Survival curves of females from five transgenic lines, F, P, X, Y, and Z, and the HWE control line following intrathoracic injection of DENV-2 or cell culture supernatant. Survival curves were performed for 15 days.

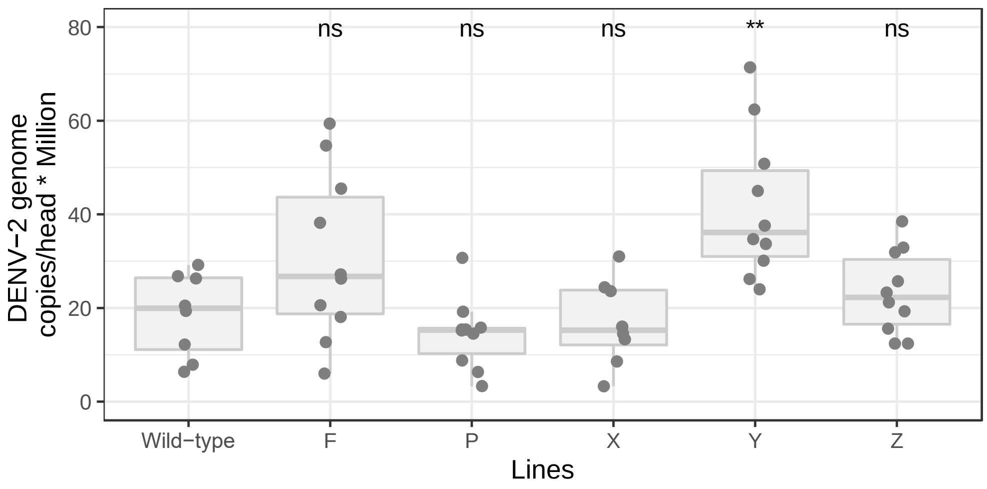

Figure S2. Median number of DENV-2 RNA copies/head (per million) detected at 15 days DPI via one-step qRT-PCR in control (HWE) females and the five transgenic lines following intrathoracic injection of the virus. The GLM procedure and the estimated marginal means (least squared mean) with a Tukey follow-up test were used for statistical analysis.

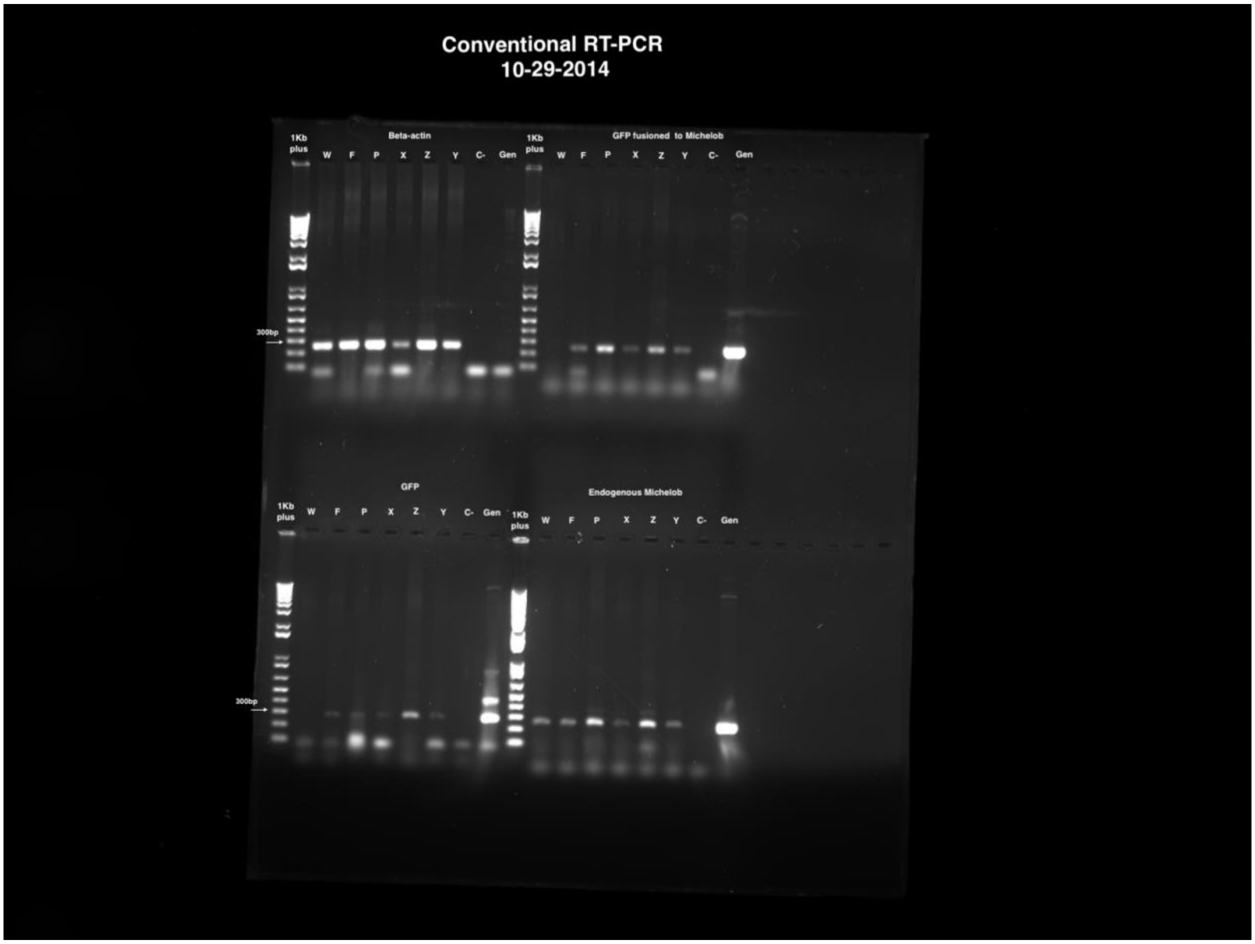

Figure S3. The original figure used to compose figure 03 of the manuscript. The upper left block (Beta-actin) corresponds to *act-1*, upper right block (GFP fused to Michlob) corresponds to eGFP_*mx*. The lower left block (GFP) corresponds to eGFP, and the lower right block (Endogenous Michelob) corresponds to *Aamx. F, P, X, Z,* and *Y* reference each VIPCD line, W corresponds to the wild-type (or WT – non-transformed line), C- corresponds to the negative control without DNA sample, and Gen represents the positive control containing genomic DNA.

Table S1. List of oligonucleotide primers used for PCR, RT-PCR, and qRT-PCR experiments.

Gene	Primer	Sequence	Fragment size	Annealing T °C
Michelob_x	M_x F	5´-AATTCCACCAACTCCTCCGT-3´	300 bp	60 °C
	M_x R	5'-GCTTGTTGCACAGCAGACAT-3'		
Actin 1	ACT F	5'-ATTGCTCCACCAGAACGTAAA-3'	300 bp	55 °C
	ACT R	5'-CAGGATTAACTTAGAAGCACT-3'		
GFP and Michelob_x	EGFP_mx F	5´-ACCACTACCTGTCGACCCAG -3´	300 bp	45 °C
	EGFP_mx R	5´-CTGCTGCTGTTGCATCATTT-3´		
